# Endogenization and excision of human herpesvirus 6 in human genomes

**DOI:** 10.1371/journal.pgen.1008915

**Published:** 2020-08-10

**Authors:** Xiaoxi Liu, Shunichi Kosugi, Rie Koide, Yoshiki Kawamura, Jumpei Ito, Hiroki Miura, Nana Matoba, Motomichi Matsuzaki, Masashi Fujita, Anselmo Jiro Kamada, Hidewaki Nakagawa, Gen Tamiya, Koichi Matsuda, Yoshinori Murakami, Michiaki Kubo, Amr Aswad, Kei Sato, Yukihide Momozawa, Jun Ohashi, Chikashi Terao, Tetsushi Yoshikawa, Nicholas F. Parrish, Yoichiro Kamatani

**Affiliations:** 1 Genome Immunobiology RIKEN Hakubi Research Team, RIKEN Cluster for Pioneering Research and RIKEN Center for Integrative Medical Sciences, Yokohama, Japan; 2 Laboratory for Statistical and Translational Genetics, RIKEN Center for Integrative Medical Sciences, Yokohama, Japan; 3 Department of Pediatrics, Fujita Health University School of Medicine, Toyoake, Japan; 4 Division of Systems Virology, Department of Infectious Disease Control, International Research Center for Infectious Diseases, Institute of Medical Science, The University of Tokyo, Tokyo, Japan; 5 Statistical Genetics Team, RIKEN Center for Advanced Intelligence Project, Tokyo, Japan; 6 Laboratory for Cancer Genomics, RIKEN Center for Integrative Medical Sciences, Yokohama, Japan; 7 Laboratory of Molecular Medicine, Institute of Medical Science, The University of Tokyo, Tokyo, Japan; 8 Laboratory for Clinical Genome Sequencing, Graduate School of Frontier Sciences, The University of Tokyo, Tokyo, Japan; 9 Division of Molecular Pathology, Institute of Medical Science, The University of Tokyo, Tokyo, Japan; 10 RIKEN Center for Integrative Medical Sciences, Yokohama, Japan; 11 Institut für Virologie, Freie Universität Berlin, Berlin, Germany; 12 Laboratory for Genotyping Development, RIKEN Center for Integrative Medical Sciences, Yokohama, Japan; 13 Department of Biological Sciences, Graduate School of Science, The University of Tokyo, Tokyo, Japan; 14 Laboratory of Complex Trait Genomics, Graduate School of Frontier Sciences, The University of Tokyo, Japan; Fred Hutchinson Cancer Research Center, UNITED STATES

## Abstract

Sequences homologous to human herpesvirus 6 (HHV-6) are integrated within the nuclear genome of about 1% of humans, but it is not clear how this came about. It is also uncertain whether integrated HHV-6 can reactivate into an infectious virus. HHV-6 integrates into telomeres, and this has recently been associated with polymorphisms affecting *MOV10L1*. *MOV10L1* is located on the subtelomere of chromosome 22q (chr22q) and is required to make PIWI-interacting RNAs (piRNAs). As piRNAs block germline integration of transposons, piRNA-mediated repression of HHV-6 integration has been proposed to explain this association. *In vitro*, recombination of the HHV-6 genome along its terminal direct repeats (DRs) leads to excision from the telomere and viral reactivation, but the expected “solo-DR scar” has not been described *in vivo*. Here we screened for integrated HHV-6 in 7,485 Japanese subjects using whole-genome sequencing (WGS). Integrated HHV-6 was associated with polymorphisms on chr22q. However, in contrast to prior work, we find that the reported *MOV10L1* polymorphism is physically linked to an ancient endogenous HHV-6A variant integrated into the telomere of chr22q in East Asians. Unexpectedly, an HHV-6B variant has also endogenized in chr22q; two endogenous HHV-6 variants at this locus thus account for 72% of all integrated HHV-6 in Japan. We also report human genomes carrying only one portion of the HHV-6B genome, a solo-DR, supporting *in vivo* excision and possible viral reactivation. Together these results explain the recently-reported association between integrated HHV-6 and *MOV10L1/*piRNAs, suggest potential exaptation of HHV-6 in its coevolution with human chr22q, and clarify the evolution and risk of reactivation of the only intact (non-retro)viral genome known to be present in human germlines.

## Author summary

Human herpesvirus 6 (HHV-6) infects most people during childhood and can reactivate later in life to contribute to diseases. The HHV-6 genome is also inherited by about 1% of people, included in the end “cap” of one of their 46 chromosomes. Little is known about how HHV-6 genomes entered human genomes, whether or not they still do, and the risk this poses for virus reactivation. We looked for HHV-6 in genome sequences from ~7,500 Japanese people. Most integrated HHV-6 variants have been co-evolving with human chromosomes for many generations. Surprisingly, in almost three-fourths of Japanese people with HHV-6 in their genome, HHV-6 is integrated in the same end of the same chromosome – 22q. Persistence of the HHV-6 genome within the short “cap” that preserves the end of chromosome 22q raises the question of whether the integrated viral sequence has taken on a useful function for this chromosome. Some human genomes harbor only one part of the HHV-6 genome–the same part that remains after experimental HHV-6 reactivation, during which most of the virus is cut out of the genome. This suggests that integration of HHV-6 into inherited human genomes is not irreversible, and possibly leads to production of infectious virus.

## Introduction

HHV-6 are betaherpesviruses and consist of two recently distinguished species, HHV-6A and HHV-6B, whose genomes share 90% nucleotide identity [1]. HHV-6 are members of the *Roseolavirus* genus, named after roseola, the clinical syndrome of fever and rash caused by primary infection by these viruses. Most people are infected with HHV-6 as infants. HHV-6B is most often responsible for primary HHV-6 infection in regions where identification of the responsible species has been performed. Although often benign, primary HHV-6 infection can lead to central nervous system disease including febrile status epilepticus [2]. HHV-6 viremia occurs in about 40% of immunosuppressed transplant recipients, in whom it can cause severe complications, including limbic encephalitis [3]. HHV-6 have also been associated with other neurological diseases including multiple sclerosis (reviewed in [4]) and Alzheimer’s disease [5]. Like other herpesviruses, HHV-6 can establish presumably life-long, latent infection. In contrast to other herpesviruses, this may require integration of the viral genome into the host chromosome [6].

Both representatives of HHV-6 were shown to have integrated into human chromosomes *in vivo* and been transmitted via the germline over 20 years ago [7,8] These early reports were controversial, because it was unclear whether the viral genome was itself inherited on the human chromosome, or if instead a conventional human chromosomal variant was inherited, and this variant conferred susceptibility to virus integration into a common integration site [9,10]. More recently, both HHV-6 species have been demonstrated to integrate into human chromosomes *in vitro* [11]. HHV-6 integrates specifically into telomeres [6]. HHV-6 genomes consist of terminal direct repeats (DR_L_ and DR_R_) flanking a unique region that encodes most of the proteins. Each DR contains two stretches of the human telomere hexameric repeat (TTAGGG)_n_, which are important for integration *in vitro* [12]. For this reason, homologous recombination has been proposed to be involved in integration, however the exact mechanism is not clear [13,14].

Chromosomally-integrated HHV-6 can recombine along the DRs *in vitro*, leading to excision of the majority of the viral genome and production of infectious virus [15–17]. This excision can leave behind one DR as a “scar” in the human chromosome. *In vivo*, one subject with X-linked SCID has been described to have become viremic with infectious HHV-6A due to reactivation of their germline-integrated HHV-6 [18], and two infants are thought to have been infected *in utero* due to reactivation of the virus integrated into their mother’s germline genome [19]. In these cases, the potential solo-DR genomic “scar” was not studied, but a single DR in a non-telomeric location has been described in one other case [20]. Excision and reactivation integrated of HHV-6 *in vivo* is thus unclear, however this question is important to understanding whether there are virus-related risks associated with this condition [21].

WGS surveys of human populations may address unanswered questions about how the HHV-6 genome has entered and exited human chromosomes. WGS of blood cells from over 8,000 subjects, mostly of European ancestry, revealed that sequences from HHV-6A or HHV-6B could be found at read depth suggesting integration into the germline genome in 0.5% of subjects [22]. In about half of these subjects, reads spanning the integration site (one end mapping to virus and the other mapping to a human chromosome) were found, consistent with chromosomal integration. From the globally diverse 1000 Genomes Project (1kGP), HHV-6 chromosomal integration was identified in 0.44% of 2,535 subjects on the basis of viral read depth [23]. In the largest study addressing this topic to date, over 140,000 subjects from China were sequenced genome-wide at low depth (0.3x) using cell-free DNA collected for non-invasive prenatal testing [24] (hereafter, Liu *et al*.). Combining HHV-6A and HHV-6B, 0.46% had viral read depths suggestive of integrated HHV-6.

Strikingly, GWAS performed on Chinese subjects with integrated HHV-6 identified a strong association with SNPs on chr22q that affect expression of a gene, *MOV10L1*, involved in production of PIWI-interacting RNAs (piRNAs). piRNAs are known to silence transposable elements (TEs), including the retrotransposons used to elongate some insect’s telomeres [25,26]. piRNAs have also been proposed to enable heritable antiviral immune memory when generated from integrated viral sequences [27,28]. Liu *et al*. interpreted the observed association to suggest that piRNAs repress HHV-6A/B integration, and that polymorphisms affecting *MOV10L1* allow for more efficient integration of HHV-6A/B during gametogenesis. We attempted to replicate the association between the piRNA pathway and integrated HHV-6 in a different cohort, using WGS data from BioBank Japan (BBJ). Our GWAS also identifies variants on chr22q that are highly associated with integrated HHV-6, however our interpretation differs substantially from that of Liu *et al*.: GWAS signals are driven by SNPs that are in linkage disequilibrium with specific “founder” integrations in subjects who share ancestry. This includes one endogenous HHV-6A variant linked to the reported *MOV10L1* SNP. We used long-read sequencing to further characterize this ancestral integration site. Unexpectedly, two independent ancestral integrations of HHV-6 into chr22q are relatively prevalent, accounting for 72% of all integrated HHV-6 in the Japanese population. We also describe molecular evidence of the recombination event that has been proposed to lead to HHV-6 reactivation from the integrated form, but lacks *in vivo* reports. In sum, we leverage human genome sequencing to address both new and long-standing clinically-relevant questions about chromosomal integration of HHV-6; the unexpected answers raise new hypotheses about these viruses’ coevolution with humans.

## Results

### Endogenization of HHV-6A on chr22q in East Asians

**Fig 1:**
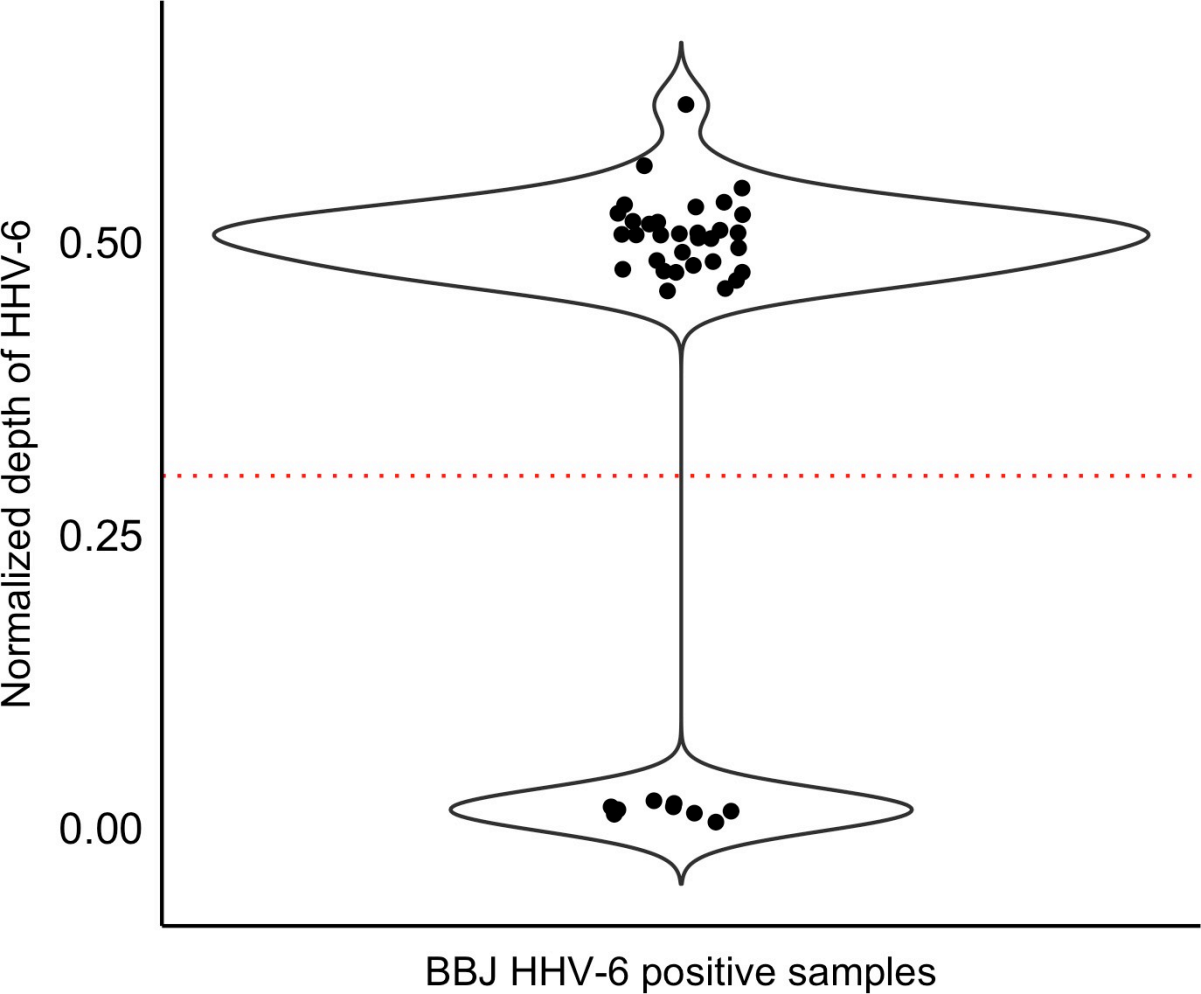
Screening for integrated HHV-6 in subjects from Biobank Japan. Unmapped WGS reads were mapped to HHV-6A (reference genome U1102). Each point represents the depth of coverage of HHV-6 for a given subject normalized by the WGS depth of that subject. Individuals with normalized depth greater than 0.3 (dashed line) were inferred to carry integrated HHV-6 (N = 32). There is a second cluster consisting of samples with low depth of coverage of HHV-6 (N = 9).

We analyzed WGS data from a total of 7,485 Japanese individuals from BBJ. We selected 32 subjects with viral read depth consistent with integrated HHV-6 based on thresholds applied in similar studies (Fig 1 and methods). None of these subjects were closer than fourth-degree relatives to each other. This suggests a prevalence of integrated HHV-6 in Japan of 0.43%. Consistent with previous results, we detected hybrid virus/human paired-end reads in 12 of these subjects [22]. However, the human chromosome reads from these mate pairs did not map uniquely to the reference genome, precluding us from assigning the site of integration [29].

**Fig 2:**
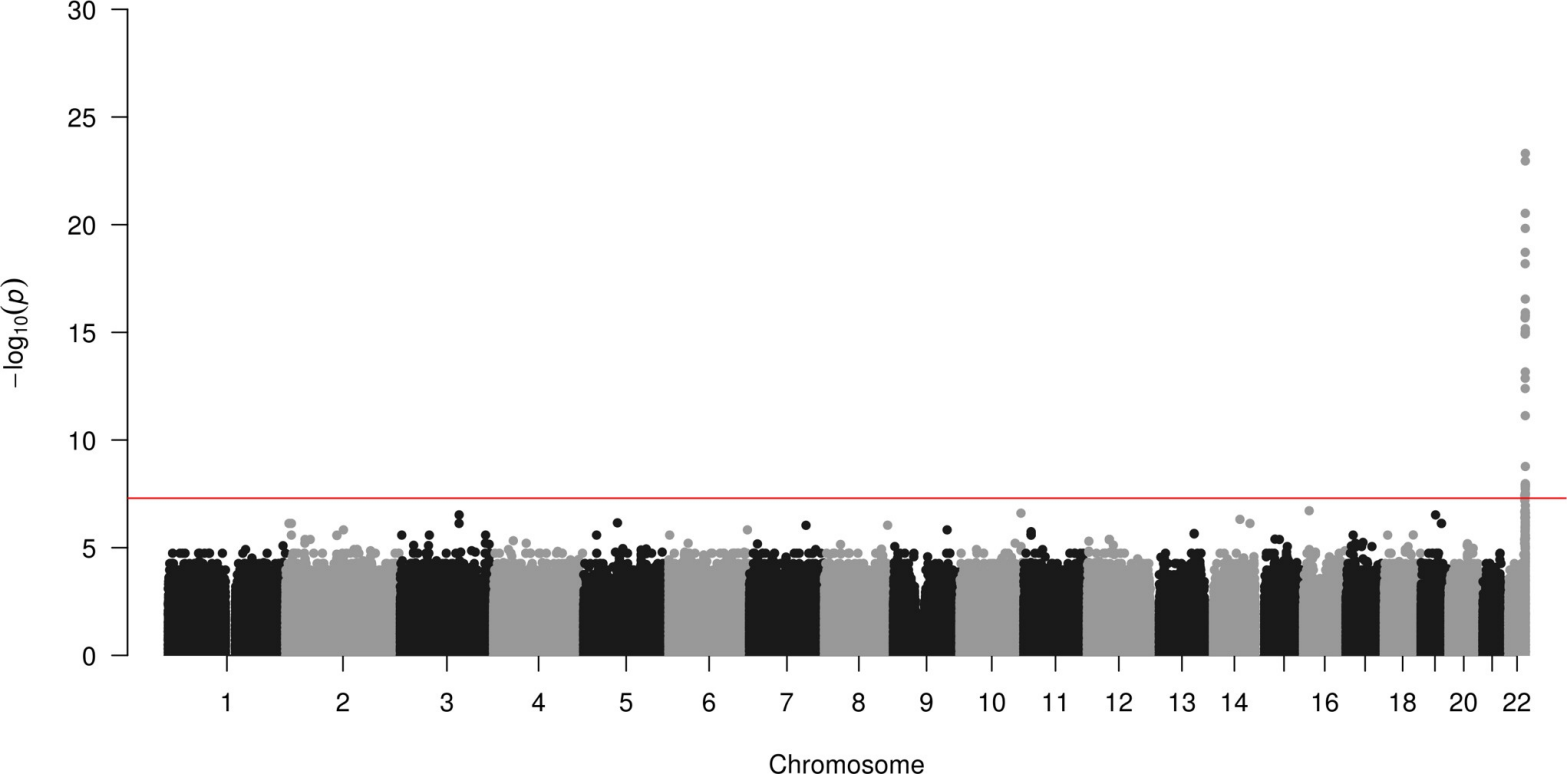
Integrated HHV-6A/B is associated with variants on chr22q. Manhattan plot presenting the *P* values for association between a variant and integrated HHV-6 (N = 32) compared to subjects who do not carry integrated HHV-6. The −log10 *P* value (Fisher’s exact test) given for each variant is plotted according to the variant’s physical position on the chromosome.

**Fig 3:**
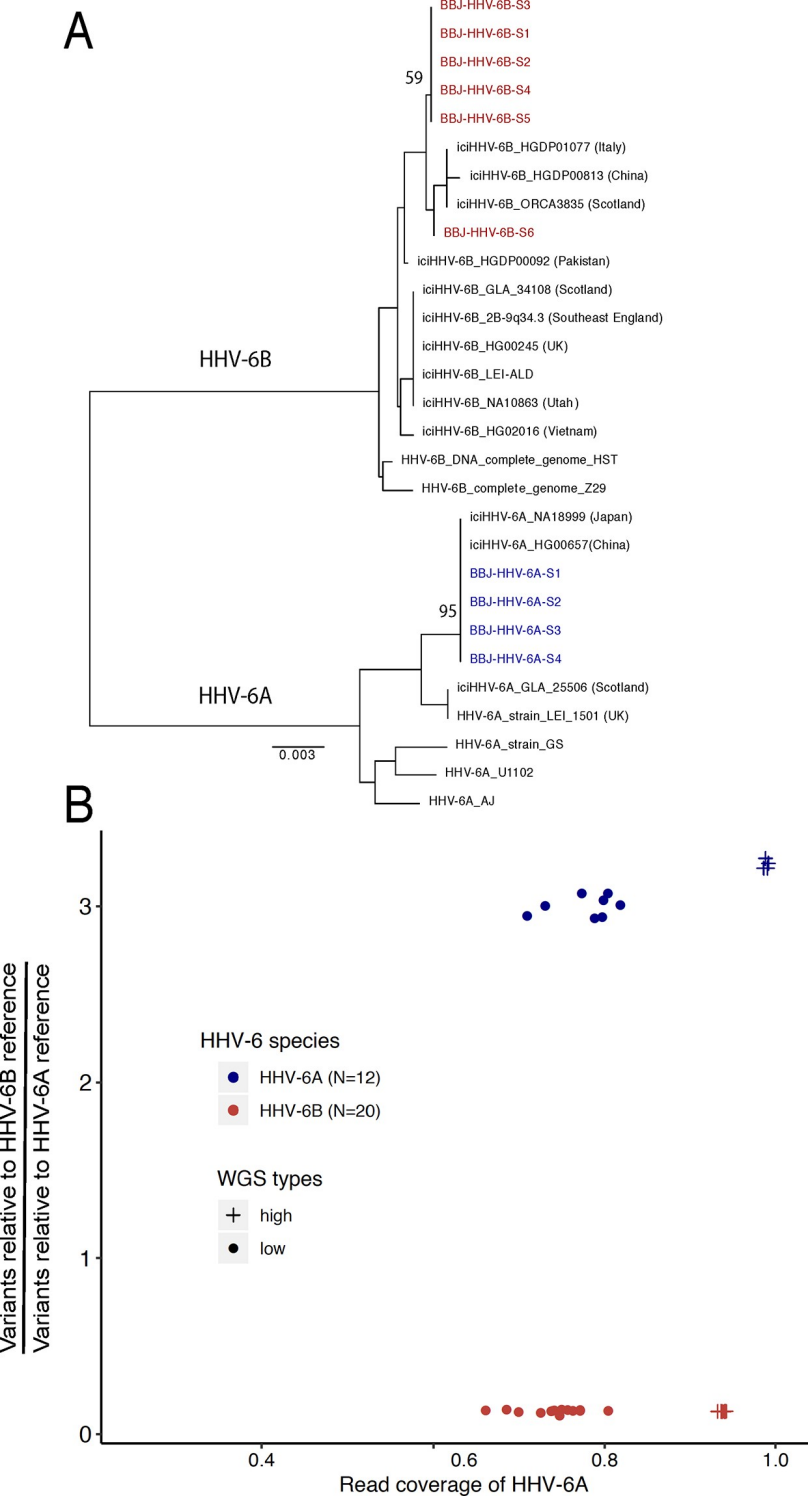
HHV-6 species is readily distinguished in subjects with both high- and low-depth WGS A) Neighbor-joining phylogenetic tree of concatenated HHV-6 viral genes U27/U43/U87 from 29 HHV-6 genomes. HHV-6A sequences from high-depth WGS samples from BioBank Japan (BBJ), colored in blue, are monophyletic and are identical to integrated HHV-6A sequences of Japanese (NA18999) and Chinese (HG00657) subjects from 1kGP. HHV-6B sequences obtained from BBJ subjects are labelled in red. Bootstrap value per 100 replicates of selected nodes is shown. The scale bar represents 0.003 substitutions per site. **B) Comparing variants and mapping coverage relative to HHV-6A and HHV-6B reference genomes distinguishes species for subjects with low-depth WGS.** The *Y* axis indicates the ratio of variants called in comparison to the HHV-6B reference versus those called in comparison to the HHV-6A reference genome. The *X* axis represents the ratio of percentage of coverage of the HHV-6B reference versus coverage of the HHV-6A reference genome. Samples determined as HHV-6A (N = 12) and HHV-6B (N = 20) are colored in blue and red, respectively. Subjects sequenced to high depth or low depth are represented by crosses and dots, respectively.

We first attempted to replicate the GWAS result reported by Liu *et al*. In spite of the small sample size (32 case subjects), we also identified polymorphisms at the distal end of chr22q that are highly associated with integrated HHV-6A/B (Fig 2). The previously reported index SNP rs73185306 was modestly associated with HHV-6A/B (*P* = 0.013, OR = 2.38); the lead SNP identified in our study is closer to the chr22q telomere and in linkage disequilibrium with more centromeric variants (S1 Table). The previous association study grouped subjects with HHV-6A and HHV-6B together, under the stated rationale that they co-occurred frequently and were potentially misclassified due to sequence homology. Using the 10- to 100-fold higher sequencing depth in our study, we first distinguished subjects with integrated HHV-6A from those with integrated HHV-6B. To do so, we extracted and concatenated three viral genes (U27/U43/U83, which show high divergence between viral species) from the BBJ subjects sequenced to high depth. A phylogenetic tree of these sequences along with reference HHV-6 sequences readily discriminated viral species. Furthermore, it showed that Japanese HHV-6A sequences were monophyletic (Fig 3A). In fact, across the 143,199 bp of non-repetitive viral genome sequence called from WGS data for all four subjects with integrated HHV-6A, there were only two unique nucleotide substitutions. Notably, U27/U43/U83 sequences from HHV-6A sequences from our study are also identical to those from integrated HHV-6A in the genome of a previously-sequenced Chinese subject (HG00657) [23], and distinct from circulating or integrated HHV-6 sequences derived from subjects in other geographic locations (Fig 3A). Some of the HHV-6B sequences were also identical across the three genes concatenated for this tree (Fig 3A). To distinguish viral species in subjects with lower sequencing depth, we calculated the ratio of the variants present in the integrated virus relative to each species’ reference genome, reasoning that there would be fewer variants called for the species to which the integrated virus belonged. Plotting these values along with those of the deeply sequenced subjects showed that, even in subjects with intermediate sequencing depth, the viral species could readily be distinguished (HHV-6A N = 12, HHV-6B N = 20; Fig 3B). Furthermore, no subjects appeared to harbor sequences from both HHV-6A and HHV-6B (Fig 3B).

**Fig 4:**
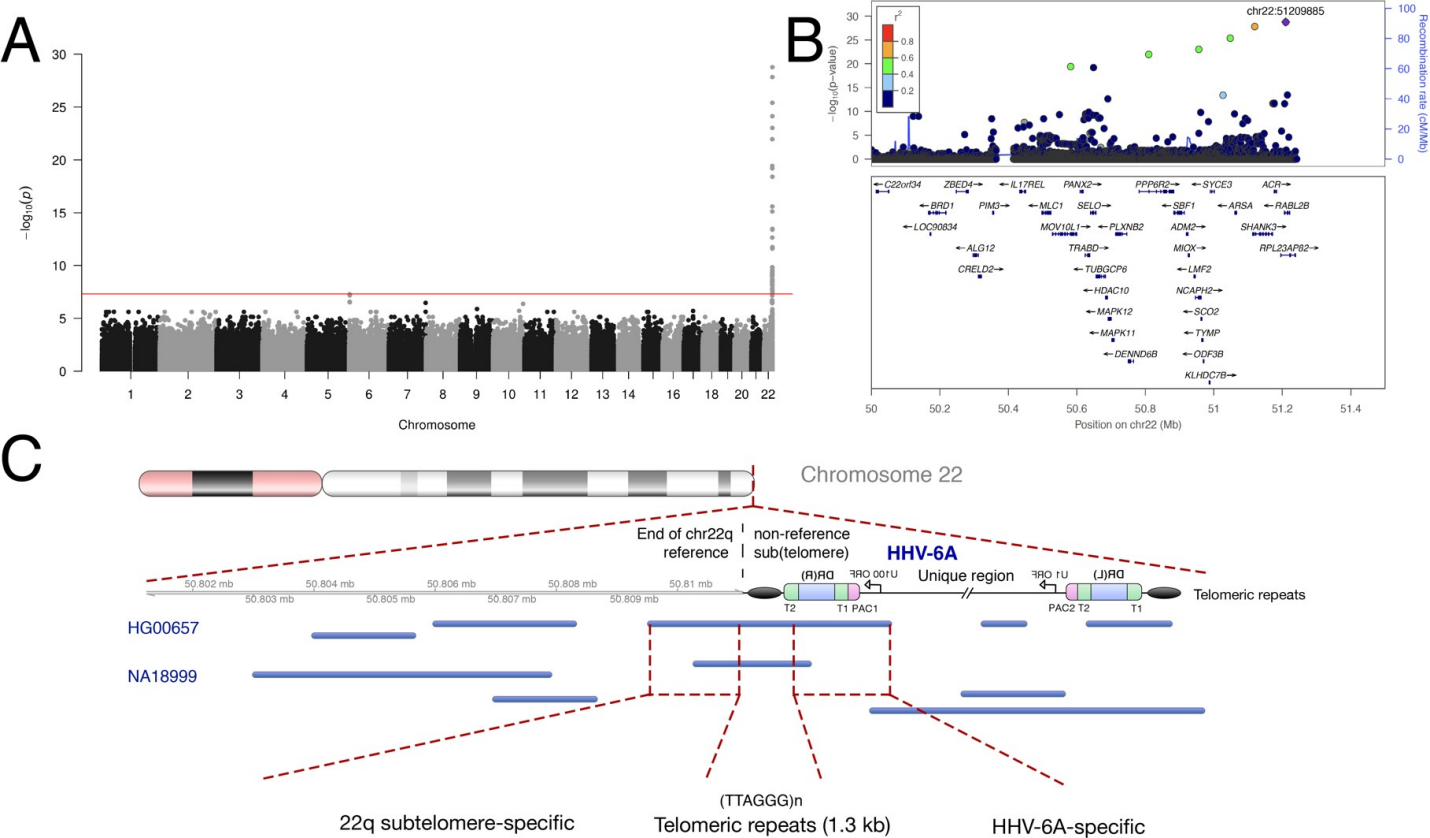
An endogenous HHV-6A variant in East Asians integrated into chr22q. **A) Manhattan plot presenting the *P* values for association between variant and integrated HHV-6A (N = 12).** The −log10 *P* value (Fisher’s exact test) given for each variant is plotted according to the variant’s physical position on the chromosome. **B) Regional association plot of the chr22q region.** The −log10 *P* value (Fisher’s exact test) for association in the GWAS of integrated HHV-6A are shown. The purple diamond symbol represents the lead variant in the region of the association. Proxies are indicated with colors determined from their pairwise r^2^ from the high-depth BBJ WGS data (red, r^2^ > 0.8; orange, r^2^ = 0.6−0.8; green, r^2^ = 0.4−0.6; blue, r^2^ = 0.2−0.4; dark blue, r^2^ < 0.2 or no information available). **C) Long-read sequencing identifies endogenous HHV-6A integration site.** Mapping of individual long sequencing reads (blue lines) to the chr22q reference sequence and to HHV-6A is depicted. Reads were obtained from lymphoblastoid cell lines derived from two subjects with integrated HHV-6A (HG00657 and NA18999) who bear the rare variant rs566665421. The reads that span the integration site are highlighted, demonstrating the integration site of HHV-6A in both subjects is the non-reference terminal heterochromatin of the q arm of chr22.

The near-identity of integrated HHV-6A viral sequences in our dataset suggested that they descended from a single integration event that increased in proportion in the population via vertical transmission. In this scenario, the SNPs associated with integrated HHV-6 variants that are identical by descent, representing a single historical event, would not necessarily be indicative of the biological factors contributing to HHV-6 integration as interpreted by Liu *et al*. Instead, these SNPs may be in linkage disequilibrium with the chromosomal integration site of the ancestral integrated HHV-6A. To test this, we repeated GWAS using only subjects with integrated HHV-6A (Fig 4A). Again, despite using only 12 case subjects, GWAS revealed a highly significant association with SNPs on chr22q, including with SNPs overlapping the locus identified by Liu *et al*. (Fig 4B and S2 Table). Notably, rs73185306 is significantly associated HHV-6A (*P* = 1.85E-05, OR = 7.36) but not HHV-6B (*P* = 0.578, OR = 0.54). From these results, we hypothesized that there was an ancient “founder” integration of HHV-6A that remains present in the telomere of chr22q in some East Asians and explains the association with *MOV10L1* in the previous study.

The lead SNP from Liu *et al*. is 780 kilobases (kb) away from the chr22q telomere, into which we suspect an ancestral integration of HHV-6A occurred. We thus hypothesized the existence of an extended haplotype comprising at least the last ~780 kb of chr22q, spanning from the telomere to this SNP. To test this, we used phase-estimated microarray data from chr22q to build a phylogenetic tree [30]. This revealed that 9 subjects with HHV-6A from BBJ clustered together with a well-supported node, indicating that they do share a haplotype on the distal end of chr22q (S1 Fig and S2 Fig). We confirmed that none of the subjects with HHV-6A from BBJ were closely related, indicating that haplotype sharing was localized to chr22q. We next tabulated rare variants highly associated with HHV-6A for subjects sequenced to high depth (Table 1). These results are concordant with the haplotype tree; the most telomeric rare variant associates perfectly with integrated HHV-6A. Some subjects carry the centromeric but not the telomeric rare variants, suggesting a crossover recombination event that replaced the telomeric portion of the haplotype; as expected, these subjects lack integrated HHV-6A. The Chinese subject (HG00657) lacks the more centromeric rare variants of the haplotype, explaining why this subject did not cluster with Japanese subjects with integrated HHV-6A (S1 Fig). However, this subject shares the most distal rare variant (rs566665421) associated with HHV-6A. This haplotype structure, along with the near identity of the viral sequences, suggests that an integrated HHV-6A variant present in both Chinese and Japanese individuals is the result of ancestral viral integration into chr22q.

**Table 1:** Rare variants in chromosome 22q subtelomeric region co-segregate with integrated HHV-6A.

		Position (centromeric to telomeric)^1^									
ID	Group^2^	22:50522524 rs73185306^3^	22:50582094 rs149078280	22:50810165	22:50874466	22:50874474	22:50956116 rs200663937	22:51048089 rs531808350	22:51058555	22:51209885 rs566665421	HHV-6 status
HHV-6A-S1	BBJ	C/T	C/T	C/G	G/GCGTTT	G/A	C/T	T/C	T/TA	A/G	HHV-6A
HHV-6A-S2	BBJ	C/T	C/T	C/G	G/GCGTTT	G/A	C/T	T/C	T/TA	A/G	HHV-6A
HHV-6A-S3	BBJ	C/T	C/T	C/G	G/GCGTTT	G/A	C/T	T/C	T/TA	A/G	HHV-6A
HHV-6A-S4	BBJ	C/T	C/T	C/G	G/GCGTTT	G/A	C/T	T/C	T/TA	A/G	HHV-6A
NA18999	1KGP (Japan)	C/T	C/T	C/G	G/GCGTTT	G/A	C/T	T/C	T/TA	A/G	HHV-6A
HG00657	1KGP (China)	C/C	C/C	C/C	G/G	G/G	C/C	T/T	T/T	A/G	HHV-6A
CTRL(JPN)-44	BBJ	C/T	C/T	C/G	G/GCGTTT	G/A	C/T	T/C	T/TA	A/A	No HHV-6 reads
CTRL(JPN)-34	BBJ	C/T	C/T	C/C	G/G	G/G	C/C	T/T	T/T	A/A	No HHV-6 reads
HHV-6B-S1	BBJ	C/C	C/C	C/C	G/G	G/G	C/C	T/T	T/T	A/A	HHV-6B
HHV-6B-S2	BBJ	C/C	C/C	C/C	G/G	G/G	C/C	T/T	T/T	A/A	HHV-6B
HHV-6B-S3	BBJ	C/C	C/C	C/C	G/G	G/G	C/C	T/T	T/T	A/A	HHV-6B
HHV-6B-S4	BBJ	C/C	C/C	C/C	G/G	G/G	C/C	T/T	T/T	A/A	HHV-6B
HHV-6B-S5	BBJ	C/T	C/C	C/C	G/G	G/G	C/C	T/T	T/T	A/A	HHV-6B
HHV-6B-S6	BBJ	C/C	C/C	C/C	G/G	G/G	C/C	T/T	T/T	A/A	HHV-6B

^1^position based on reference Hg38 and dbSNP ID, if applicable

^2^BBJ, Biobank Japan; 1KGP, from 1000 Genomes Project subjects with integrated HHV-6, Telford *et al*. [23]

^3^index SNP from Liu *et al*. [24]

We considered that linkage disequilibrium (LD) between this integrated HHV-6A genome and the SNP reported by Liu *et al*. might explain their association result. However, if that were the case, we reasoned that the most telomeric variant (rs566665421), rather than one in *MOV10L1*, would be most highly associated SNP with HHV-6A/B integration. We checked this site in a database providing summary statistics from Liu *et al*. (https://db.cngb.org/cmdb/), but genotyping data is not available. Therefore, we speculate that the low sequencing depth used for the previous study of integrated HHV-6 in East Asians precluded accurate genotyping of variants more closely linked to this trait, prevented recognition of a large LD block in the region of the association, and obscured evidence of shared ancestry of the integrated HHV-6A allele, resulting in identification of an index SNP distant from the “causal” HHV-6A variant present in the telomere.

To confirm that the HHV-6A-linked haplotype is physically linked to telomere-integrated HHV-6, we performed long-read sequencing. Only two subjects with publicly-available genotypes carry the rare variant rs566665421: the Chinese subject mentioned above (HG00657) and one Japanese subject (NA18999) [31]. We hypothesized that these subjects harbor the same endogenous HHV-6A variant integrated into chr22q. We obtained lymphoblastoid cell lines derived from these subjects and performed long-read sequencing. This yielded individual reads that mapped to both HHV-6A and to the subtelomere of chr22q (Fig 4C). There are approximately 1.3 kb of hexameric repeats between the viral DR_R_ and the last base pair of the chr22q reference sequence.

To confirm physical linkage of integrated HHV-6A with chr22q in another way, we used DNA samples from 6 Japanese subjects previously identified to have inherited chromosomally integrated HHV-6A. In these subjects, the integration site had previously been mapped using fluorescent in situ hybridization (FISH) to chr22q [32]. Including HG00657 and NA18999, we sequenced SNPs in eight subjects with integrated HHV-6A mapped to chr22q (S3 Table). The SNP reported by Liu *et al*. was present in six of these subjects, whereas all carried rs566665421. Thus both FISH mapping and direct sequencing localize an endogenous HHV-6A variant shared by Japanese and Chinese subjects to the telomere of chr22q, linked to a shared extended haplotype.

When this HHV-6A integration event occurred and when the allele entered the Japanese population may be informative about its prevalence in other populations. To estimate integration timing, we assumed that mutations arising in the HHV-6A genome after integration accumulate at the same rate as other chromosomal mutations (see methods). The number of polymorphisms present in the six deeply-sequenced HHV-6A genomes suggests that the virus integrated 30,556 years ago (95% CI 15,253–54,672) assuming a generation time of 25 years (S3 Fig). Considering polymorphisms in the HHV-6A genomes present only in Japanese subjects gives an estimate of 14,881 years (95% CI 4,832 to 34,727). Of the 11 polymorphisms observed, each observed in a single subject, 3 were in non-coding regions, 4 were missense, and 4 were synonymous. Next, we estimated how long the HHV-6A-linked haplotype has been recombining in the Japanese population. We modeled this using a simple deterministic equation based on the decay of linkage disequilibrium (LD) between integrated HHV-6A and a linked marker allele (rs149078280-T) [33]. This estimate suggested a much more recent introduction to Japan, roughly 875 years ago (CI 250–2,350 years; S3 Fig). Although the models differ in how recently this haplotype arrived to Japan, both models suggest that this endogenous HHV-6A allele existed in other East Asian populations prior to entering the Japanese population, perhaps during the Jomon period (14,000 to 300 BCE). Consistent with this interpretation, we observed that SNPs linked with the viral integration are present in Northeastern Asian populations at a similar frequency to these SNPs in the Japanese population (NARD Database, https://nard.macrogen.com).

### Endogenization of HHV-6B on chr22q

**Fig 5:**
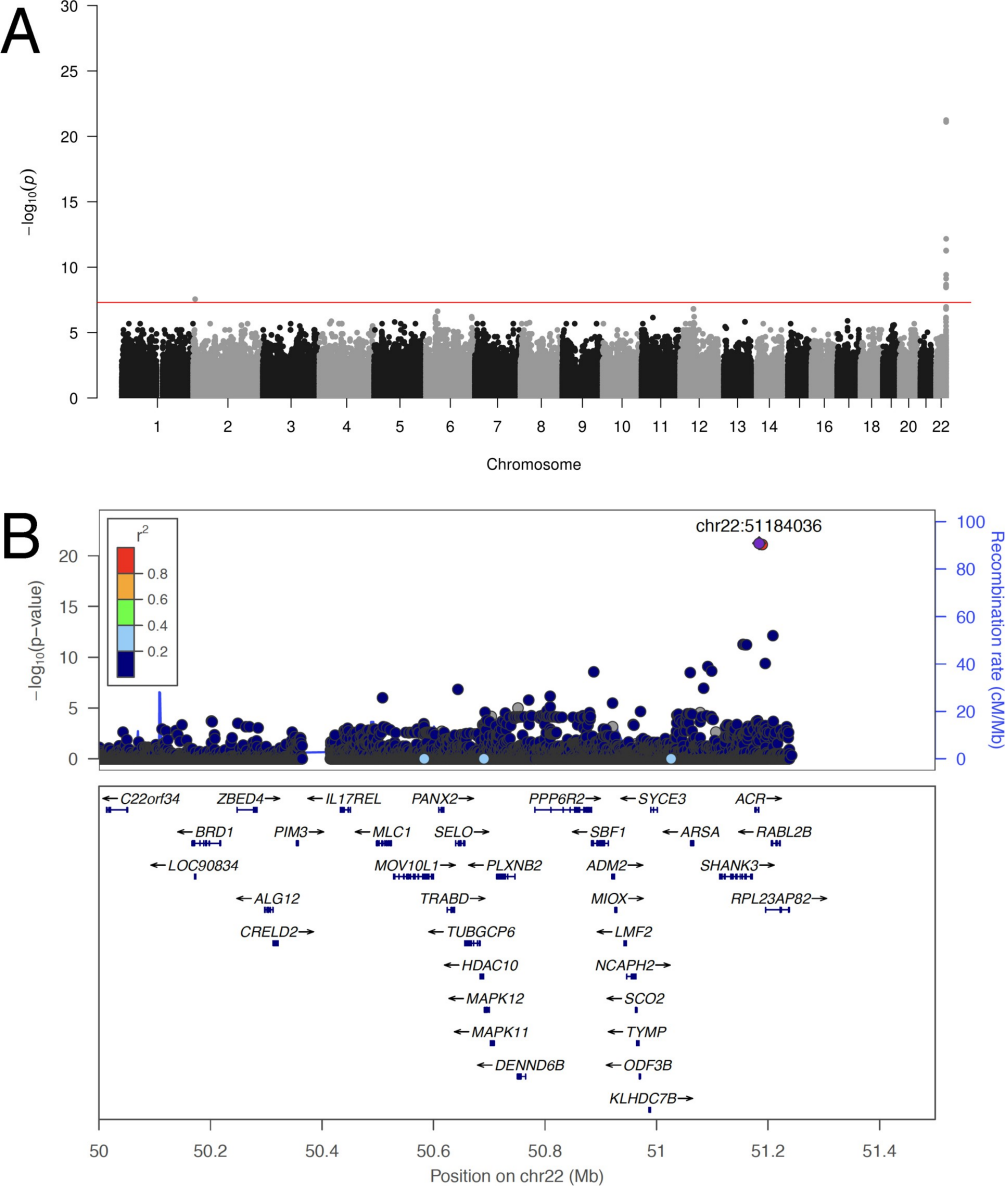
A prevalent endogenous HHV-6B variant integrated into chr22q. **A) Manhattan plot presenting the P values for association between variants and clonal integrated HHV-6B (N = 20).** The −log10 *P* value (Fisher’s exact test) given for each variant is plotted according to the variant’s physical position on the chromosome. **B) Regional association plot of the 22q region.** The −log10 *P* value (Fisher’s exact test) for association in the GWAS of integrated HHV-6A are shown. The purple diamond symbol represents the lead variant in the region of the association. Proxies are indicated with colors determined from their pairwise r^2^ from the high-depth BBJ WGS data (red, r^2^ > 0.8; orange, r^2^ = 0.6−0.8; green, r^2^ = 0.4−0.6; blue, r^2^ = 0.2−0.4; dark blue, r^2^ < 0.2 or no information available).

Subjects whose HHV-6B sequences were nearly identical also clustered on chr22q in the haplotype analysis (S4 Fig), unexpectedly suggesting another variant with shared ancestry. We performed GWAS using 11 subjects with this clonal integrated HHV-6B variant (Fig 5A). Consistent with integration into the same chromosome arm as the endogenous HHV-6A variant described above, this integrated HHV-6B variant is also associated with SNPs on chr22q (S4 Table). The haplotype linked to this viral genome is more common than that on which HHV-6A is integrated, with fewer significantly associated variants extending into the subtelomere (Fig 5B). To confirm that this GWAS result reflects physical linkage, we obtained DNA from three subjects previously identified to have integrated HHV-6B mapped by FISH to chr22 [32]. Sanger sequencing of the most highly associated variant (chr22:51184036) revealed that all were heterozygous for the minor allele, and none carried the rs73185306 variant (S5 Table). Together, these results show that in the Japanese population, two different chr22q haplotypes are associated with the majority of integrated HHV-6 – one with HHV-6A and the other with HHV-6B – and that shared chr22q haplotypes correspond with shared, clonal integrated HHV-6 sequences, representing endogenous HHV-6.

### Excision of HHV-6B from the genome

We next analyzed subjects with reads mapping to HHV-6A at a depth below the threshold used to infer germline integration. We analyzed the coverage of the viral genome in these subjects and compared to those with integrated HHV-6 described above. This revealed two distinct coverage patterns: subjects with reads mapped across the entire HHV-6 genome, and subjects with reads mapped to the DR region only (S5 Fig). This partial coverage pattern was not observed in any subjects previously inferred to carry integrated HHV-6A/B. The depth of reads covering the DR region was lower in subjects who lack U region-mapped reads, suggesting that a single copy of the DR is present in these subjects. Sequencing coverage of the DR region in these subjects terminated abruptly adjacent to the viral genome packaging sequences (Pac1/Pac2). Notably, this solo-DR configuration has been previously proposed as the molecular signature of recombination along the DRs, shown *in vitro* to lead to viral reactivation [17].

BBJ WGS data was derived from DNA extracted from nucleated blood cells. We hypothesized that clonal expansion of a hematopoietic lineage in which recombination and excision of integrated HHV-6 had occurred could result in detection of only DR sequences from blood-derived DNA. If this were the case, the entire HHV-6 genome could be present in other cells, but at low abundance in the blood. To indirectly assess this, we obtained additional blood-derived DNA from these subjects and performed digital droplet PCR using primers for both the DR region and a well-conserved U region (S6 Fig). As a control, we used DNA from subjects from whom the entire HHV-6 viral genome was detected by WGS. There was no PCR-detectable evidence of low-level U-region integration observed for subjects whose WGS reads mapped only to the DR region. All subjects with a solo-DR integration were determined to be of subtype HHV-6B using the species-specific DR probe used for digital droplet PCR (S6 Fig). This result argued against mosaicism as the explanation for WGS reads mapping only to the DR region.

**Fig 6:**
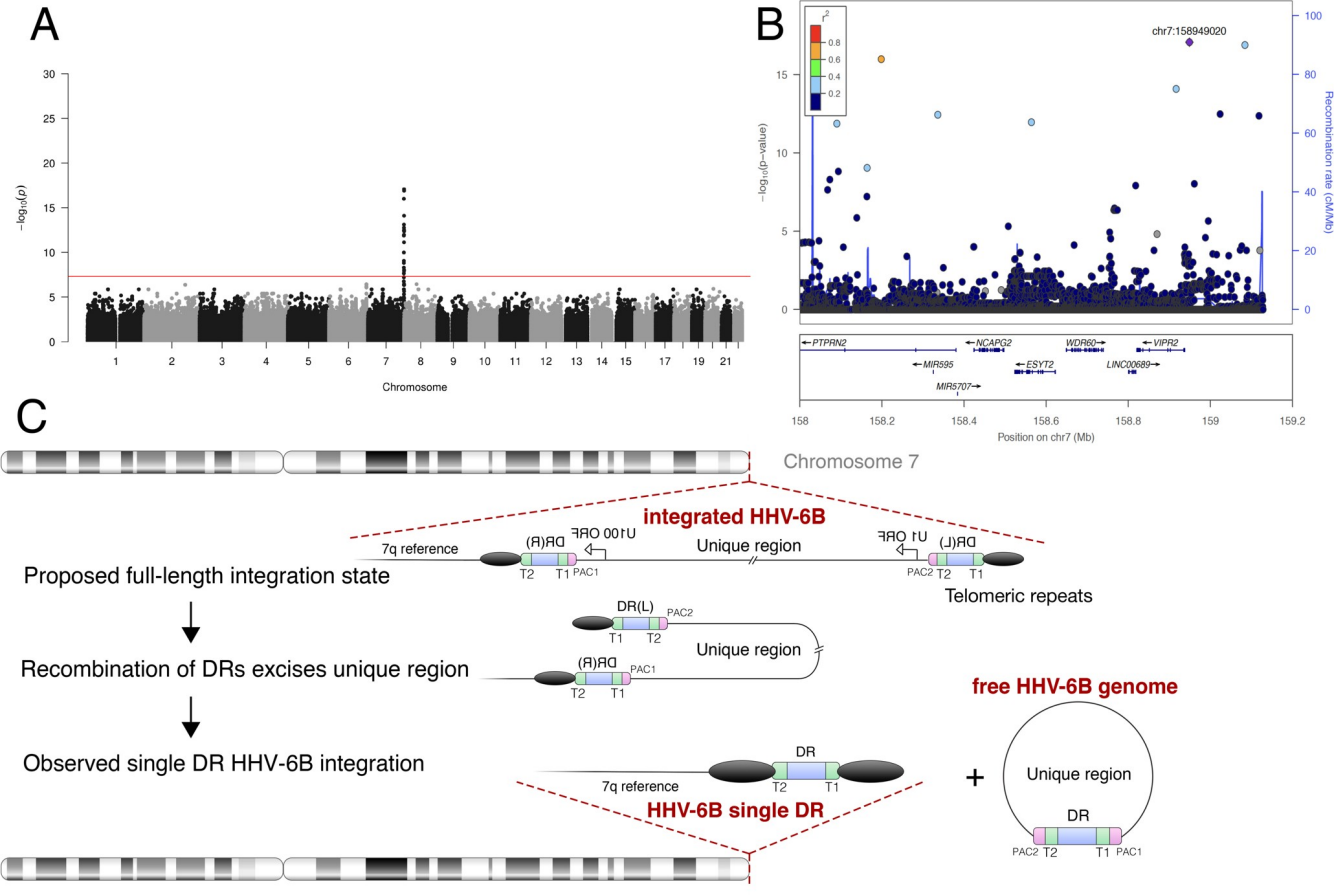
Recombination and excision of HHV-6B from chromosome 7q. **A) Manhattan plot from GWAS of subjects bearing HHV-6B solo-DR integration (N = 9).** The −log10 *P* value (Fisher’s exact test) given for each variant is plotted according to the variant’s physical position on the chromosome. **B) Regional association plot of the 7q region.** The −log10 *P* value (Fisher’s exact test) for association in the GWAS of integrated HHV-6A are shown. The purple diamond symbol represents the lead variant in the region of the association. Proxies are indicated with colors determined from their pairwise r^2^ from the high-depth BBJ WGS data (red, r^2^ > 0.8; orange, r^2^ = 0.6−0.8; green, r^2^ = 0.4−0.6; blue, r^2^ = 0.2−0.4; dark blue, r^2^ < 0.2 or no information available). **C) Model of HHV-6 recombination and excision resulting in the observed integrated solo-DR.** Schematic of the proposed germline recombination event (after [34]) leading to excision of the majority of integrated HHV-6B sequence resulting in the integrated solo-DR form.

We next performed phylogenetic analysis of integrated HHV-6B DR regions from all deeply-sequenced subjects, including four with solo-DR integration, to further clarify the evolution of the integrated solo-DRs (S7 Fig). Three of the DR sequences from subjects with solo-DR integration were identical, and a fourth varied from these at two sites. Based on this result, we hypothesized that the solo-DR sequence mostly shared by these subjects could potentially reflect a single historical recombination event. To further clarify this point, we performed GWAS using 9 subjects bearing solo-DR HHV-6B integration (Fig 6). Consistent with a single founder integration into the human chromosome and excision of the majority of the viral genome in the germline (Fig 6C), subjects bearing solo-DR HHV-6B integration often shared telomere-proximal SNPs on 7q.

## Discussion

Large WGS datasets offer a unique opportunity to study the human virome and human-virus coevolution. This is especially true in the case of integrated HHV-6, which results from unconventional virus-to-host horizontal gene transfer. However, even the prevalence of this interesting condition remains uncertain. For example, the commonly-cited prevalence of 1% in the global population may be an overestimate of the prevalence in healthy subjects, perhaps driven by studies analyzing patient samples, among which the prevalence appears to around 2% [35]. In that context, it is notable that less than 1% of the subjects in the diverse populations, some healthy (e.g. 1kGP), and others disease-enriched (e.g. BBJ), screen positive for integrated HHV-6 using WGS. Our observation that some subjects retain only a portion of the integrated viral genome, a solo-DR region, has not been reported by previous WGS-based screens. It is unclear whether such subjects have been detected in screens using other methods; if so, they were grouped together with subjects harboring full-length HHV-6 integration. While this form is not a potential source of viral reactivation from the host genome, the DR region encodes genes as well as microRNAs which may influence host or exogenous viral gene expression [36]. Considering the existence of solo-DR integration as a distinct category of integrated HHV-6 is important for future studies on the implications of HHV-6 integration for human health.

In addition, the incidence of HHV-6 integration has remained unclear in the 20 years since this phenomenon was first described. However, careful studies using even small cohorts have been able to infer that ancestral integrations could account for much of integrated HHV-6 [37]. Recent work in Europeans, focusing on viral rather than human genetic diversity, has also suggested that integrated HHV-6 can reflect ancient, ancestral integrations [38]. Analysis of integrated HHV-6 in Japan clearly shows that integrated HHV-6 most often reflects ancestral, rather than incident, events in this population. Thus the evolutionary history of integrated HHV-6, which may be more precisely described as “endogenous HHV-6” in examples for which multigenerational germline inheritance is demonstrated, influences the interpretation of any associated human chromosomal SNPs [24]. Considering the incidence and prevalence of chromosomally-integrated forms of HHV-6 is also important for properly interpreting the association of HHV-6 with phenotype and disease [5,21,39].

We provide molecular resolution of the relatively common East Asian endogenous HHV-6A integration breakpoint, advancing the study of HHV-6 from a human genetic and paleovirological perspective. Unexpectedly, we demonstrated that the majority of both integrated HHV-6A and HHV-6B in the Japanese population is located at the same cytogenetic locus, the telomere of chr22q. Further study of this phenomenon is needed. The hypothesis that polymorphisms of this subtelomere prevent piRNAs from blocking HHV-6 integration, as they have been shown to do for other mobile genetic elements that can invade the germline, was a provocative one. In known examples of piRNA-guided silencing of TEs, integration of the element into a genomic locus that produces piRNAs is required [40]. In some species, the telomeres are in fact piRNA-generating loci. For example, piRNAs are produced from telomere-integrated retrotransposons in flies and silkworms, as well as the large TEs known as terminons in the telomeres of rotifers [41]. “piRNA-like” RNAs have been reported to derive from mouse telomeres [42]. Whether telomere-integrated HHV-6 can act as a template to produce piRNAs, potentially protecting the germline from subsequent HHV-6 integration, remains to be tested. However, our work suggests that the proposed mechanism to explain the association between *MOV10L1* and integrated HHV-6, i.e. that piRNAs usually block HHV-6 integration but do not do so efficiently in subjects with a SNP affecting *MOV10L1*, is not supported by a number of independent integration events attributable to this SNP. At least in Japan, there seems to have been only one such integration; this same endogenous virus also exists in China and likely arrived to Japan from continental Asia.

What then explains the relatively prevalent endogenous HHV-6 variants, from both HHV-6A and HHV-6B, in the telomere of chr22q in Japanese subjects? We cannot exclude stochasticity, i.e. that it reflects two independent founder effects. If we assume that such a founder effect would be equally likely to be observed for HHV-6 integrated into any chromosome arm, the likelihood of observing both on the same chromosome arm is one in 46. Chr22q has been reported to carry the penultimate shortest human telomere, longer only than that of 17p [43]. Chromosome 17p has notably been shown to harbor multiple HHV-6 integrations in Europeans [10,38]. These loci are both associated with subtelomere deletion syndromes, chr22q13 deletion syndrome and 17p13 monosomy [44,45], which likely result from critical telomere shortening. In many non-human organisms, mobile DNA can extend telomeres (reviewed in [46]). More work is needed to test whether human chromosomes with short telomeres benefit from carrying endogenous HHV-6 [47].

We used two methods to estimate the timing of integration of the endogenous HHV-6A variant prevalent in East Asian populations. The estimates suggest that HHV-6A integrated into the chromosome of an ancestral continental East Asian and arrived later in Japan. This model is consistent with human phylogeography and the observed distribution of rare alleles present on this haplotype in other populations. However, the confidence intervals of the two estimates of arrival to Japan do not overlap. The estimate based on mutation accumulation, which has been used previously to provide reasonable estimates of HHV-6 integration timing, places the arrival of this allele to Japan in the more distant past than that based on recombination. Perhaps this HHV-6A variant is accumulating mutations more rapidly than expected for chromosomal sequences. However, while the sample size is small, the observed polymorphisms in the integrated HHV-6A sequence do not evidence selection for nonsynonymous and potentially virus-inactivating mutations. Another possibility is that the haplotype is recombining less frequently than expected. The latter could be interpreted as consistent with the HHV-6A-linked haplotype evolving under positive selection in Japan, although an effect of the viral genome on homolog pairing and synapsis cannot be excluded. Our current study is underpowered to further address this intriguing possibility.

We described the molecular signature of HHV-6 excision, via recombination, from its position in the telomere for the first time. As extensively characterized *in vitro*, this event can enable viral reactivation, with potential production of infectious virus. We observed the solo-DR form in nine subjects in BBJ, about 30% of all subjects with some form of integrated HHV-6B sequence. This could reflect as few as one historical excision event in the germline, however these data provide evidence, previously lacking, that the process does occur *in vivo*, not only *in vitro*. While confirming that the risk of excision and thus potential reactivation of integrated HHV-6B is real, full-length integrated HHV-6 genomes with two DRs identical to the solo-DR were not observed, and thus the likelihood of the excision event remains difficult to quantify. Studies with somatic tissues sampled from many sites may be useful for this purpose [48]. Furthermore, while recombination resulting in a solo-DR “scar” is one of the proposed routes of excision and reactivation, another involves “scarless” excision due to recombination of telomeric repeats flanking the virus genome [34]. WGS analysis is unable to infer this type of excision. Our results support caution in using cells and tissues from subjects bearing integrated HHV-6 for transplantation: upon immunosuppression, exposure to HHV-6 excised from donor cells may be harmful [49,50]. More generally, these results support the concept that HHV-6 excision from destabilized telomeric heterochromatin, for example in the aged, may contribute to human disease [5,21,39]. With data from completed or ongoing population WGS projects, a global assessment of integrated HHV-6 prevalence, evolution, and association with disease should soon be possible. In addition, understanding any immune responses engendered by endogenous HHV-6, either humoral [48] or cell-intrinsic [51], are relevant to understanding the biological significance of this phenomenon.

## Methods

### Screening of HHV-6 carriers based on WGS

A total of 7,485 WGS samples were obtained from the BBJ project [52,53]. Read alignment to the human reference genome hs37d5 and variant calling were previously performed for 3,256 high-depth (15-35x) WGS samples as described elsewhere [54]. Additionally, 4,229 intermediate-depth (3.5x) WGS were analyzed with Genomes on the Cloud (GotCloud) pipeline [55]. From each BAM file, we extracted unmapped reads. We required that both paired-end reads were unmapped. We realigned unmapped reads to the HHV-6A genome (KJ123690.1) using the BWA-MEM algorithm (BWA version: 0.7.13) [56]. Read depth was measured as the mode of per-base read depth across the length of the viral reference genome. The threshold of 30% depth relative to WGS depth of coverage was chosen in order to capture those with inconsistent mapping or some degree of acquired somatic mosaicism, but exclude those with viremia, which has resulted in 10-1000x lower coverage in other studies [22,24].

### Kinship analysis or genetic correlation matrix

We evaluated genetic relatedness between subjects based on genotypes of common variants across the genome by plink software [57]. We excluded the HLA region and restricted variants with minor allele frequency more than 5% and not in linkage disequilibrium with other variants (r^2^ > 0.2). PI_HAT, the proportion of identity by descent (IBD) defined as probability (IBD = 2)+0.5×probability (IBD = 1), was computed to determine genetic relatedness (PI_HAT> 0.25).

### GWAS of HHV-6

We performed variant joint-calling for high-depth WGS (N = 3,262) by aggregating individual gVCF with GATK following the current recommended best practice. Briefly, variant QC was conducted for samples in two subsets 1) samples sequenced at 30X (N = 1,292), variants that meet any of the following criteria (1) DP < 5, (2) GQ < 20, or (3) DP > 60, and GQ < 95 were removed; 2) samples sequenced at 15x (N = 1,964), variants that meet any of the following criteria (1) DP < 2, (2) GQ < 20 were removed. We phased the resulting genomes for use as a reference panel using SHAPEIT2 and imputed the variants of low-depth WGS using IMPUTE2 [58]. The final variant set include both high-depth and low-depth WGS. We performed exact test to compare the allele frequency between integrated HHV-6 and non-carriers for all variants using Plink (version 1.9). 5 × 10^−8^ is used as threshold to define the genome-wide significance levels.

### Viral phylogenetic analysis

We reconstructed the HHV-6 viral genome of 10 subjects with high HHV-6 read depth who have been sequenced at high-depth (4 HHV-6A and 6 HHV-6B). First, reads mapped to HHV-6A (KJ123690.1) were further aligned against the integrated HHV-6A genome derived from a Japanese individual NA18999 (GenBank Accession number: KY316047.1) using BWA-MEM. Based on the alignment, variants calling was performed using freebayes (version: v1.2.0-2-g29c4002) with parameters ploidy = 1 and min-alternate-fraction = 0.8 [59]. We generated subject-specific integrated HHV-6 viral sequences by applying the resulting variants to the KY316047.1 reference genome using the FastaAlternateReferenceMaker function in the Genome Analysis Toolkit (GATK) v3.7. These 10 samples, together with previously reported chromosomally-integrated or nonintegrated HHV-6 genomes from the literature (N = 19), were used for viral phylogenetic analysis. We extracted and concatenated sequences of 3 viral genes (U27, U43, U83) from each sample. Trees were built using the neighbor-joining algorithm with 1,000 times bootstrap using MEGA7 software [60]. The HHV-6 genomes used in this analysis and their GenBank Accession numbers are shown (S6 Table). We used the same method for phylogenetic analysis of integrated HHV-6B and others by concatenating shared variant sites that were called in all subjects.

### Haplotype phasing for variants in chr22q region

We extracted 174 variants within chr22q sub-telomeric region for high HHV-6 read-depth individuals (N = 32) and unrelated HHV-6-negative subjects (N = 100) from the microarray dataset of BBJ as described previously [61]. Genotype data of two subjects with integrated HHV-6 for whom sequence data was available through the 1000 genome project (NA18999 and HG00657) were also added [31]. A telomeric variant was appended to reflect presence or absence of integrated HHV-6 based on WGS. We used PHASE to infer the individual haplotypes, and subsequently generated the phylogenic tree based on neighbor-joining (NJ) method with MEGA (version 7) [62].

### Sanger sequencing of subjects with low-depth WGS or FISH-mapped integrated HHV-6

Sanger sequencing was conducted to genotype four variant sites including rs73185306, rs149078280, rs566665421 and chr22_51184036_C_G for BBJ subjects with integrated HHV-6 sequenced by low-depth WGS and an additional 9 Japanese subjects with integrated HHV-6 previously mapped by FISH. The PCR primers and sequencing primer sequences are provided (S7 Table). Briefly, we used 10 ng of genomic DNA for PCR amplification. Purified PCR products were sequenced on an Applied Biosystems Genetic Analyzer 3130 (Thermo Fisher Scientific, MA, USA) with BigDye Terminator v3.1.

### Estimation of the age of integrated HHV-6A

The first method to estimate the timing of the shared HHV-6A integration is based on the assumption that the observed Chinese and Japanese integrated HHV-6A genomes derived from a single integration event, and after being integrated the mutation rate of the integrated HHV-6A genome is the same as that of other human chromosomal DNA. By considering variants unique to one or more variants but absent in the consensus of all variants (interpreted to be *de novo* mutations arising after integration), we estimated the expected age and 95% CI according to the Poisson distribution. The human mutation rate is estimated at 1.2*10^-8 per site per generation and we assume 25 years between generations [63]. We excluded the repeat-rich, 2-copy DR regions and considered only variants arising in a 140 kb unique (U) genic region. We performed joint calling using FreeBayes for 4 BBJ integrated HHV-6A and HG00657 and NA18999, and subsequently filtered out unique mutations and visually confirmed the mutations by IGV.

The second method is based on the decay of linkage disequilibrium (LD) between integrated HHV-6A and a linked marker allele (rs149078280-T). The population frequencies of integrated HHV-6A allele, rs149078280-T allele, and haplotype bearing these two alleles are denoted by *p*, *q*, and *h*. The recombination rate between two loci is denoted by *c*. In this setting, *h* in the next generation is given by

$$h_{t+1}=(1-c)h_{t}+cpq.$$

The extent of LD between the two loci (*δ*) is characterized by the fraction of rs149078280-T allele among integrated HHV-6A-bearing chromosomes (i.e., *h*/*p*). Dividing the above equation by *p*, we obtain

$$\delta_{t+1}=(1-c)\delta_{t}+cq.$$

At the time of integration (*t* = 0), *δ* is 1 and then decreased exponentially at a rate *c* to *q* as generation passes. Since integrated HHV-6A is rare, the hitchhiking effect of integrated HHV-6A on the frequency of rs149078280-T (*q*) is very limited. It is therefore assumed that *q* has been constant since the integration. Solving the above recurrence equation, the current *δ* is given by

$$\delta_{t}={\left(1-q)(1-c\right)}^{t}+q.$$

Solving this for *t*, we obtain

$$t=\frac{\ln\left(\delta -q\right)-\ln\left(1-q\right)}{\ln\left(1-c\right)}.$$

The observed values of *δ* and *q* were 0.666 (= 8/12) and 0.000313. In this study the position of rs149078280 on chromosome 22 (73.29267 cM) was retrieved from genetic maps for the 1000 Genomes Project variants (https://github.com/joepickrell/1000-genomes-genetic-maps). The integrated HHV-6A genome is located telomeric to the terminal nucleotide present in the reference sequence of chr22q. Therefore we used a value of 74.10956 cM, which represents the distance from rs149078280 to the most telomeric informative allele on this chromosome. This value derives from subtelomeric markers, not the telomere itself; the genetic distance is thus a conservative underestimation of the actual distance. Accordingly, the recombination rate (*c*) between integrated HHV-6A and rs149078280 was assumed to be 0.00817 (i.e., 0.01x[74.10956–73.29267]). Putting δ, *q*, and *c* into the above equation, *t* is 35 (generations). If we assume the human generation time is 25 years, this corresponds to approximately 875 years ago. The estimated age is dependent on the numbers of haplotypes observed (i.e., observed haplotype frequencies). The haplotype frequencies used here are estimated based on a random set of 14,970 chromosomes from Japanese subjects. The numbers of integrated HHV-6A _ rs149078280-T, integrated HHV-6A _ rs149078280-C, without integrated HHV-6A _ rs149078280-T, and without integrated HHV-6A _ rs149078280-C haplotypes were 9, 3, 6, and 14,952 respectively. To obtain an empirical bootstrap confidence interval, we generated 100,000 bootstrap samples, each of size 14,970, that satisfied the condition that four different haplotypes were observed and *δ*-*q*>0. The 95% confidence interval of age was 10–94 generations (250–2,350 years) (S2B Fig).

### Nanopore long read sequencing

We obtained lymphoblastoid cell lines (LCLs) of HG00657 and NA18999 from the Coriell Cell Repositories and cultured them according to the protocol provided. We extracted high molecular weight (HMW) DNA using Gentra Puregene Cell Kit (Qiagen, Hilden, Germany) from 5 × 10^6^ cultured cells. DNA was quantified with a Qubit fluorometer (Invitrogen, US) and 1 μg of HMW DNA was used to construct the DNA library using Nanopore ligation sequencing kit SQK-LSK109 (Oxford Nanopore Technologies, ONT, Oxford, UK). We loaded the library into R9.4 flow cell (ONT) and subsequently conducted sequencing on a MinION (ONT) sequencer. Base calling for the MinION raw sequencing data was done on a MinIT (ONT) via MinKNOW software (ONT). We used minimap2 to align the reads against a customized hg19 reference genome in which the HHV-6A reference genome has been added as a decoy sequence.

### Digital droplet PCR (ddPCR)

We conducted ddPCR to determine the existence of DR and U region of HHV-6 and to distinguish the 6A/6B subtype for subjects with low depth of coverage of HHV-6 (N = 9), and control subjects carrying integrated HHV-6A (N = 2) or HHV-6B (N = 2) from BBJ. We used a primer/probe set for HHV-6 U57 gene and RPP30 (autosomal control) as previously described [64]. For the DR region, we designed common primers and species-specific probe for 6A and 6B by identifying a region of species-specific variation using an alignment of reference HHV-6 sequences and those identified in our study. Primer and probe information is provided (S8 Table). To prepare the ddPCR reaction mix, 10 μl of 2× ddPCR Supermix for Probes (Bio-Rad, Hercules, CA), 1 μl of each 20× primer-probe mix (18μM each PCR primer, 5μM probe), and 15 ng genomic DNA in a final volume of 20μl. The reaction mixture was loaded onto a DG8 cartridge (Bio-Rad) with 70μl of droplet generation oil (Bio-Rad) and processed in the Droplet Generator (Bio-Rad). After droplet generation, 40 μl droplets were transferred into a 96-well plate and proceed to thermal cycling with the following conditions: 95°C for 10 minutes, 94°C for 30 seconds and 60°C for 1 minute for 40 cycles and ending at 98°C for 10 minutes. After amplification, the droplets were read by the Droplet Reader (Bio-Rad). QuantaSoft analysis software (V1.3.2.0) was used for data analysis and quantified copy number of target molecules per μl was obtained and analyzed.

## Supporting information

S1 Fig Neighbor-joining phylogenetic tree of phased chromosome 22q subtelomeric haplotypes with HHV-6A cluster highlighted. SNPs of 22q subtelomere were phased to obtain estimates of individual haplotypes for 32 subjects with high HHV-6-mapping read depth, 100 control subjects without HHV-6-mapping reads, and subjects NA18999 and HG00657 (data from 1kGP). Branches containing the clustered HHV-6A-associated haplotype is shown expanded (see S2 Fig for fully expanded tree). Chinese subject HG00657 is highlighted with a black triangle. The HHV-6 sequence carried by this individual is shared with BBJ HHV-6A subjects (Fig 3), yet the shared 22q subtelomeric haplotype is lost except for the most telomeric rare variant (Table 1) Bootstrap value per 100 replicates of selected nodes is shown. 174 SNPs were phased.
(TIFF)

S2 Fig Neighbor-joining phylogenetic tree of phased chromosome 22q subtelomeric haplotypes from BBJ. SNPs of 22q subtelomere were phased to obtain estimates of individual haplotypes for 32 subjects with high HHV-6-mapping read depth, 100 control subjects without HHV-6-mapping reads, and subjects NA18999 and HG00657 (data from 1kGP). Lines and labels mark haplotype sharing among subjects with integrated HHV-6. Branches of this tree are selectively collapsed in S1 Fig and S4B Fig.
(EPS)

S3 Fig Estimated dating of East Asian endogenous HHV-6A integration. **A) Simulated age of integrated HHV-6A in Japan based on accumulated mutations.** We estimated the integration age by assuming that the mutation rate of the integrated HHV-6 genome is same as other human chromosomal sequences and each generation is 25 years. The blue line simulates the expected accumulation of mutations over time, the *x*-axis indicates the true age, *y*-axis indicates the expected age calculated based on number of observed mutations, and the gray area represents the 95% CI of the expected age**. B) Empirical distribution of the age of endogenous HHV-6A in Japan based on recombination.** Histogram showing the distribution of predicted age in generations (*x*-axis) of the endogenous HHV-6A allele obtained by 100,000 bootstrap samples.
(TIFF)

S4 Fig A clonal endogenous HHV-6B variant is present in Japanese subjects with a shared chromosome 22q haplotype. **A) Clonal integrated HHV-6B evidenced by phylogenetic analysis.** Joint-calling of variants was performed for BBJ subjects with integrated HHV-6B of both high and low depth (N = 20). 44 variant sites which were called in all subjects were selected and concatenated for phylogenic tree analysis using the maximum likelihood method. Subjects clustering by chr22q haplotype analysis, shown below, are bolded. Bootstrap value per 100 replicates of selected nodes is shown. The scale bar represents 0.20 substitutions per site**. B) Neighbor-joining phylogenetic tree of phased 22q subtelomeric haplotypes with clonal HHV-6B cluster highlighted.** SNPs of 22q subtelomere were phased to obtain estimates of individual haplotypes for 32 subjects with high HHV-6-mapping read depth, 100 control subjects without HHV-6-mapping reads, and subjects NA18999 and HG00657 (data from 1kGP). Branches containing clustered clonal HHV-6B associated haplotypes are shown expanded; Bootstrap value per 100 replicates of selected nodes is shown. 174 SNPs were phased.
(TIFF)

S5 Fig A subset of subjects with integration of an HHV-6B solo-DR. **A) Depth in subjects with reads mapping only to the DR region is half that of those with reads mapping across the entire viral genome.** We summarized the depth of coverage in 1kb sliding window across the HHV-6B genome (x axis), the read depth from subjects in one of two groups is plotted (y axis). The average depth of subjects meeting the described threshold to infer integrated HHV-6 from high-depth WGS (N = 10; 4 integrated HHV-6A and 6 HHV-6B) are shown in blue. The average depth of subjects with HHV-6-mapping reads below the threshold from high-depth WGS are shown in red (N = 4). Subjects failing to reach the threshold to infer integration of intact HHV-6 also produce reads of depth consistent with germline chromosomal integration of a portion of the HHV-6 genome, the DR region. A decoy HHV-6B reference genome with the DR(R) removed (which is identical in sequence to DR(L)) was used for mapping and calculation. **B) Zoomed view of coverage of the DR region.** Comparison of the depth of reads mapping to the DR region between those with full and solo-DR integration suggests that a single copy of the DR region remains in the latter. Pac1 and Pac2 sequences important for viral genome packaging are highlighted in red. T1 and T2 are telomere repeat sequences. DR1 and DR6 are spliced open reading frames present in viral genome annotation ID #NC_000898.
(TIFF)

S6 Fig Digital droplet PCR to estimate the copy number of DR and U regions. **A) DR copy number normalized by RPP30.** The bar plot shows the normalized copy number (CN) for each individual determined by 6A/6B specific DR probe. NC, negative control. **B) U57 copy number normalized by RPP30.** The bar plot shows the normalized copy number (CN) for each individual determined by 6A/6B specific U57 probe. NC, negative control.
(TIFF)

S7 Fig Phylogenetic analysis of integrated HHV-6B based on the DR region. We performed joint-calling of variants in the DR region for BBJ subjects. We considered subjects sequenced at high-depth (N = 10) to exclude the possibility of inaccurately-called variants in the repeat-rich DR region in subjects with low read depth. A total of 237 variant sites in DR region which were called in all subjects were selected and concatenated, then used to generate a phylogenic tree using the maximum likelihood method. Bootstrap value per 100 replicates of selected nodes is shown. The scale bar represents 0.005 substitutions per site.
(TIFF)

S1 Table Genome-wide significant variants from GWAS of integrated HHV-6A/B. (XLSX)

S2 Table Genome-wide significant variants from GWAS of integrated HHV-6A. (XLSX)

S3 Table Sanger sequencing of additional Japanese subjects with integrated HHV-6A mapped by FISH to chromosome 22q. (XLSX)

S4 Table Genome-wide significant variants from GWAS of clonal integrated HHV-6B. (XLSX)

S5 Table Sanger sequencing of additional Japanese subjects with integrated HHV-6B mapped by FISH to chromosome 22q. (XLSX)

S6 Table Additional HHV-6 genome sequences included in current study. (XLSX)

S7 Table Primers used for Sanger sequencing. (XLSX)

S8 Table Primers and probes used for digital droplet PCR. (XLSX)
