# Supplementary figures and images for: Endogenization and excision of human herpesvirus 6 in human genomes
Source: PLoS Genet. 2020 Aug 10;16(8):e1008915. doi: 10.1371/journal.pgen.1008915 (PMC7444522; doi:10.1371/journal.pgen.1008915)

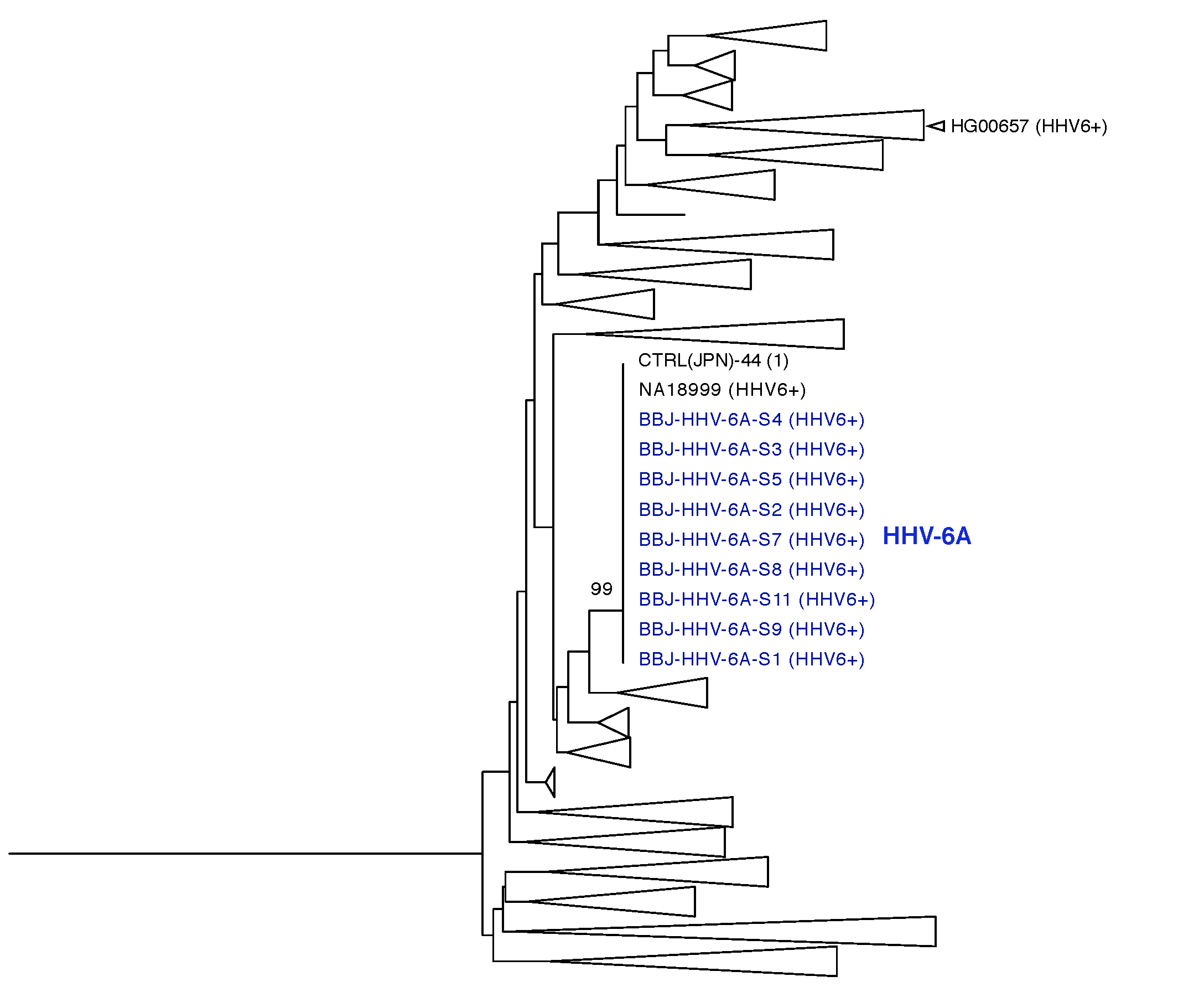

Supplement: S1 Fig — SNPs of 22q subtelomere were phased to obtain estimates of individual haplotypes for 32 subjects with high HHV-6-mapping read depth, 100 control subjects without HHV-6-mapping reads, and subjects NA18999 and HG00657 (data from 1kGP). Branches containing the clustered HHV-6A-associated haplotype is shown expanded (see S2 Fig for fully expanded tree). Chinese subject HG00657 is highlighted with a black triangle. The HHV-6 sequence carried by this individual is shared with BBJ HHV-6A subjects (Fig 3), yet the shared 22q subtelomeric haplotype is lost except for the most telomeric rare variant (Table 1) Bootstrap value per 100 replicates of selected nodes is shown. 174 SNPs were phased. (TIFF) [file pgen.1008915.s001.tiff]

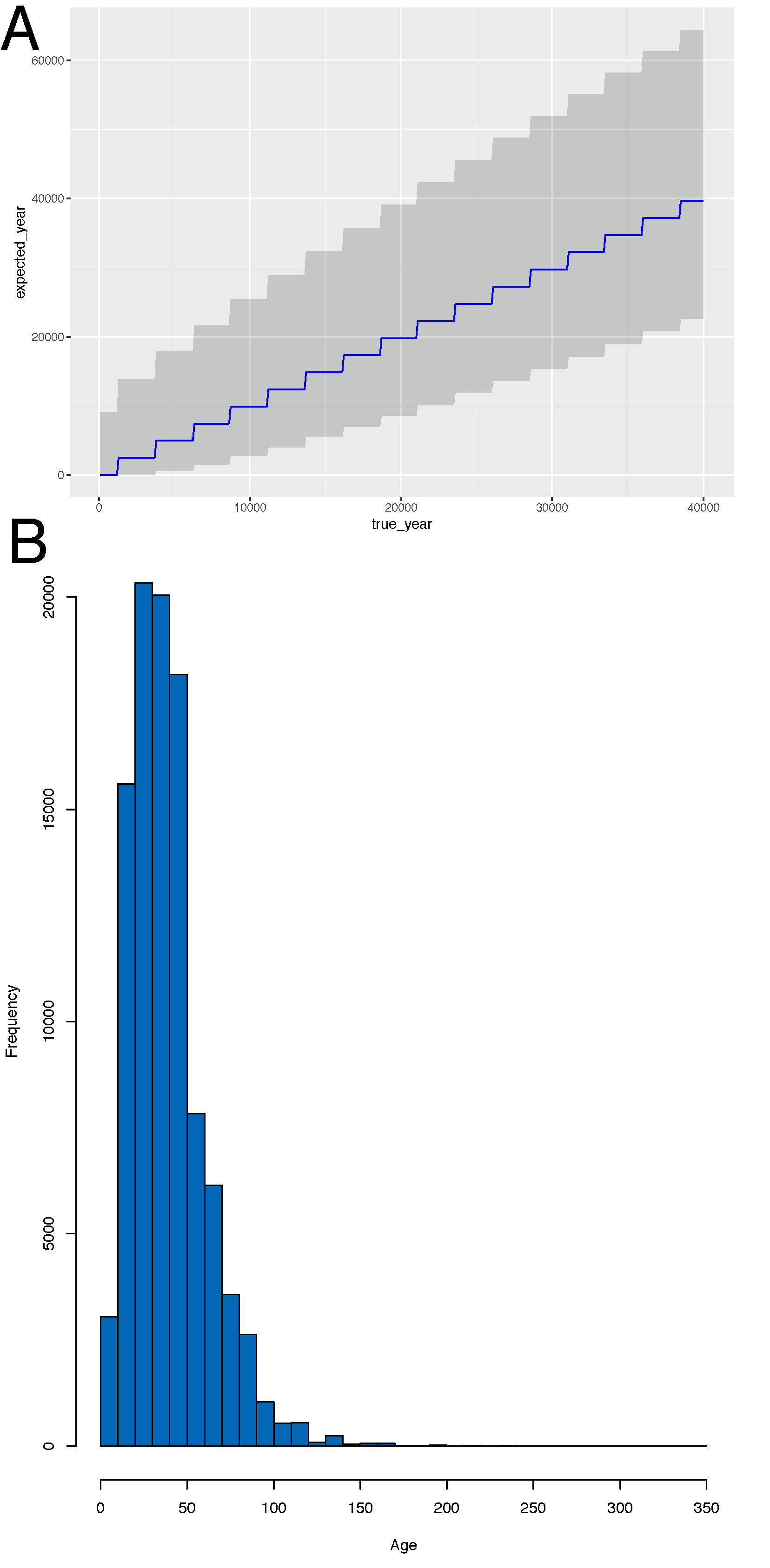

Supplement: S3 Fig — A) Simulated age of integrated HHV-6A in Japan based on accumulated mutations. We estimated the integration age by assuming that the mutation rate of the integrated HHV-6 genome is same as other human chromosomal sequences and each generation is 25 years. The blue line simulates the expected accumulation of mutations over time, the x-axis indicates the true age, y-axis indicates the expected age calculated based on number of observed mutations, and the gray area represents the 95% CI of the expected age. B) Empirical distribution of the age of endogenous HHV-6A in Japan based on recombination. Histogram showing the distribution of predicted age in generations (x-axis) of the endogenous HHV-6A allele obtained by 100,000 bootstrap samples. (TIFF) [file pgen.1008915.s003.tiff]

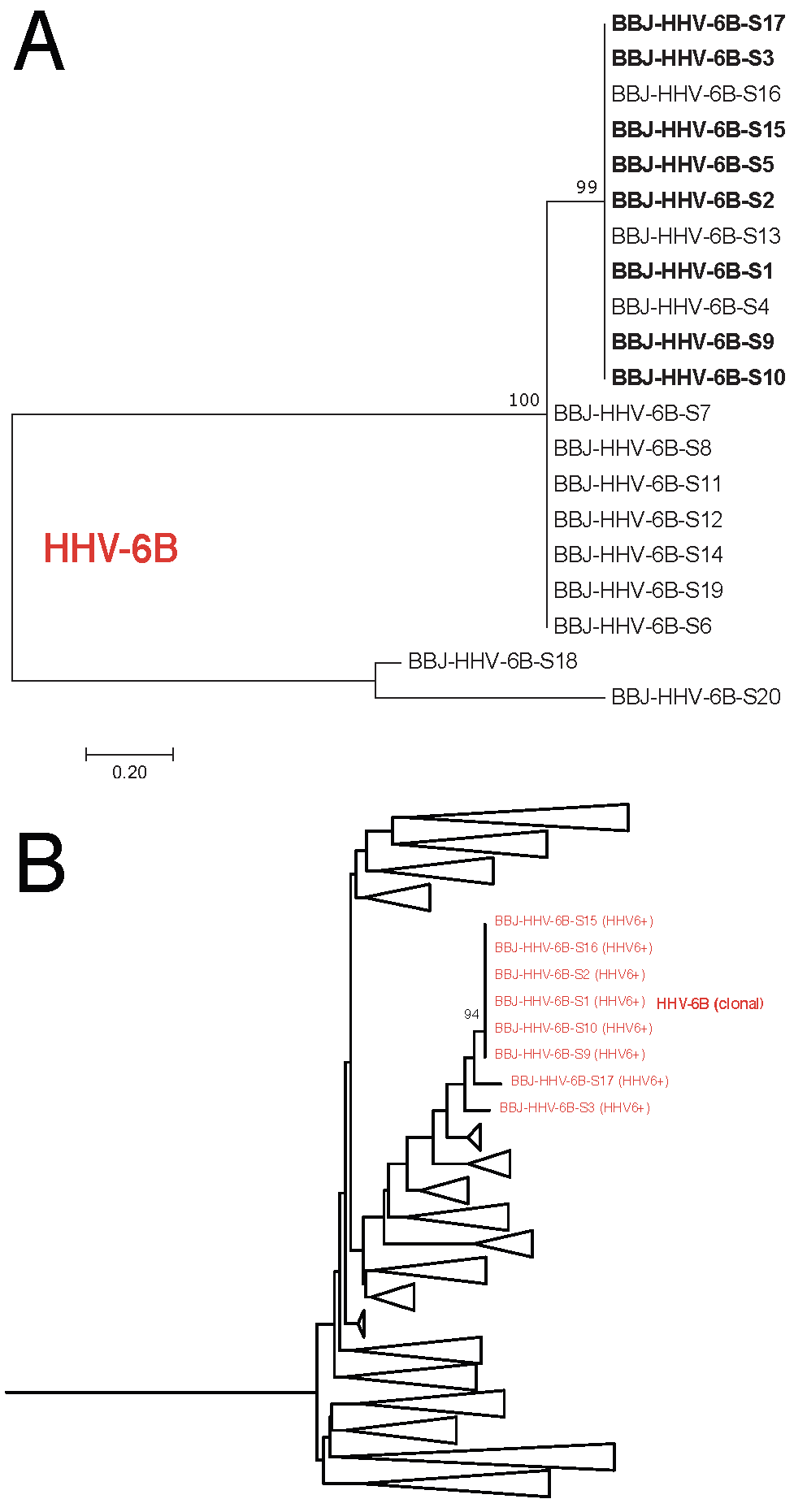

Supplement: S4 Fig — A) Clonal integrated HHV-6B evidenced by phylogenetic analysis. Joint-calling of variants was performed for BBJ subjects with integrated HHV-6B of both high and low depth (N = 20). 44 variant sites which were called in all subjects were selected and concatenated for phylogenic tree analysis using the maximum likelihood method. Subjects clustering by chr22q haplotype analysis, shown below, are bolded. Bootstrap value per 100 replicates of selected nodes is shown. The scale bar represents 0.20 substitutions per site. B) Neighbor-joining phylogenetic tree of phased 22q subtelomeric haplotypes with clonal HHV-6B cluster highlighted. SNPs of 22q subtelomere were phased to obtain estimates of individual haplotypes for 32 subjects with high HHV-6-mapping read depth, 100 control subjects without HHV-6-mapping reads, and subjects NA18999 and HG00657 (data from 1kGP). Branches containing clustered clonal HHV-6B associated haplotypes are shown expanded; Bootstrap value per 100 replicates of selected nodes is shown. 174 SNPs were phased. (TIFF) [file pgen.1008915.s004.tiff]

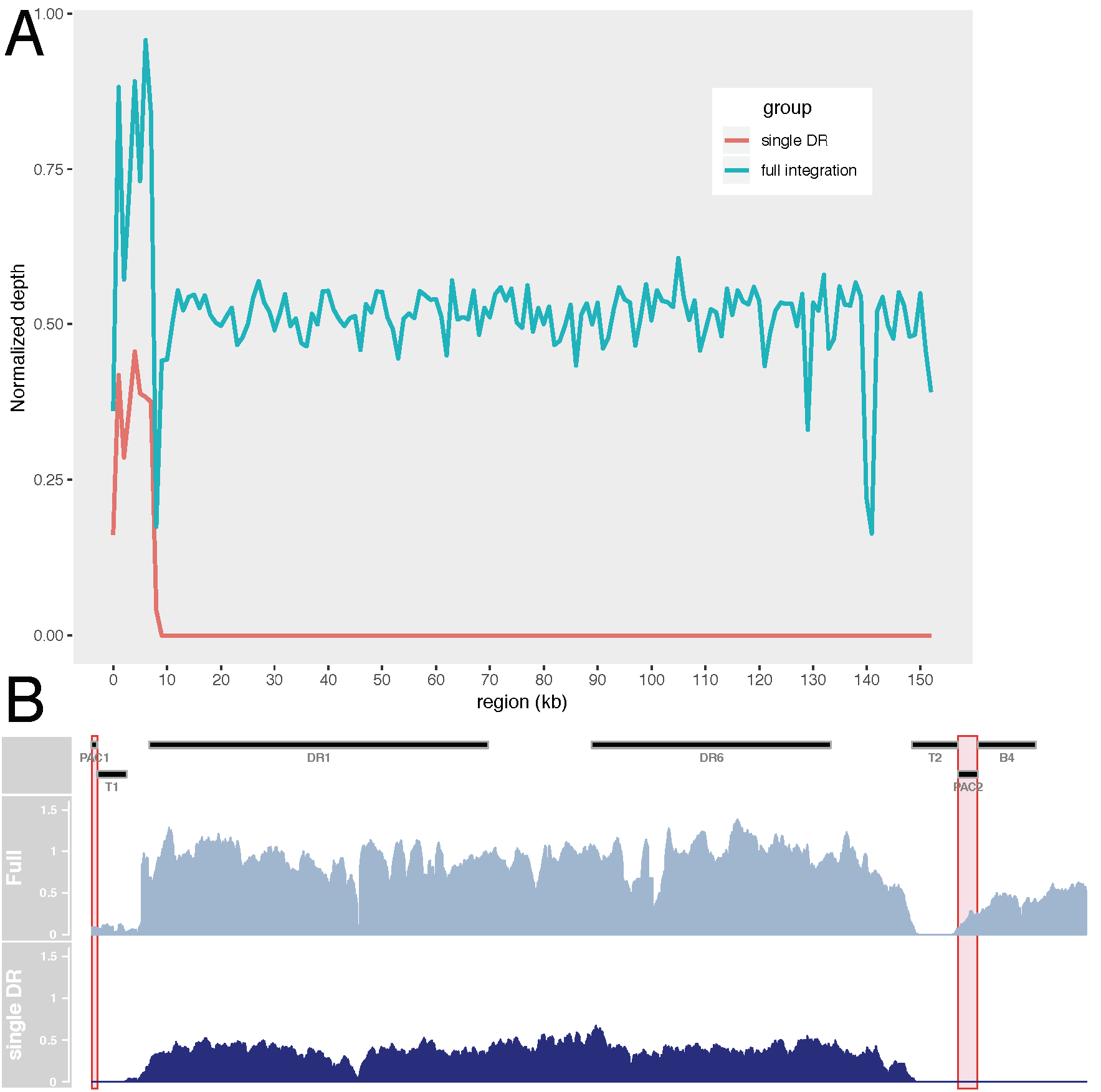

Supplement: S5 Fig — A) Depth in subjects with reads mapping only to the DR region is half that of those with reads mapping across the entire viral genome. We summarized the depth of coverage in 1kb sliding window across the HHV-6B genome (x axis), the read depth from subjects in one of two groups is plotted (y axis). The average depth of subjects meeting the described threshold to infer integrated HHV-6 from high-depth WGS (N = 10; 4 integrated HHV-6A and 6 HHV-6B) are shown in blue. The average depth of subjects with HHV-6-mapping reads below the threshold from high-depth WGS are shown in red (N = 4). Subjects failing to reach the threshold to infer integration of intact HHV-6 also produce reads of depth consistent with germline chromosomal integration of a portion of the HHV-6 genome, the DR region. A decoy HHV-6B reference genome with the DR(R) removed (which is identical in sequence to DR(L)) was used for mapping and calculation. B) Zoomed view of coverage of the DR region. Comparison of the depth of reads mapping to the DR region between those with full and solo-DR integration suggests that a single copy of the DR region remains in the latter. Pac1 and Pac2 sequences important for viral genome packaging are highlighted in red. T1 and T2 are telomere repeat sequences. DR1 and DR6 are spliced open reading frames present in viral genome annotation ID #NC_000898. (TIFF) [file pgen.1008915.s005.tiff]

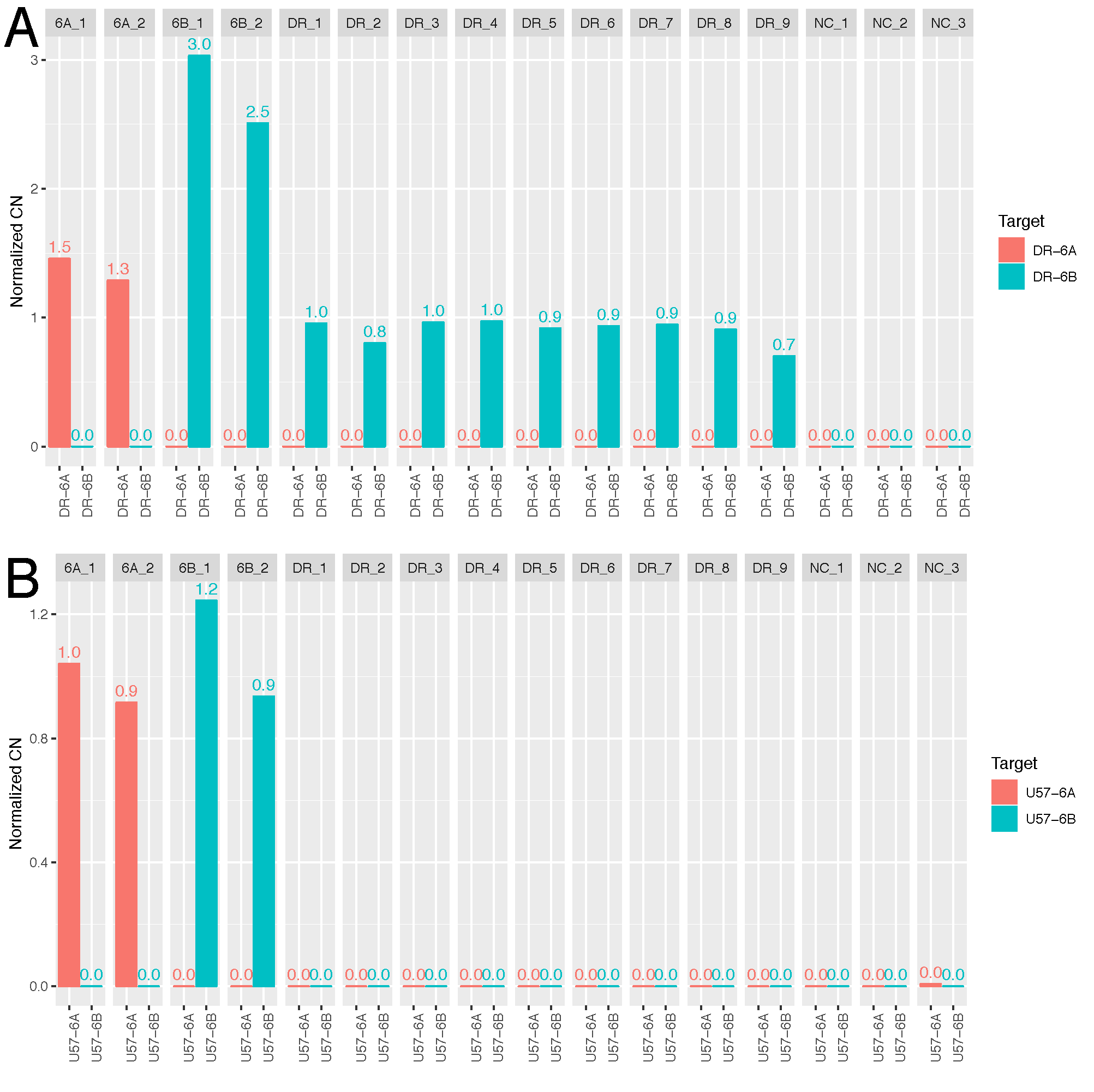

Supplement: S6 Fig — A) DR copy number normalized by RPP30. The bar plot shows the normalized copy number (CN) for each individual determined by 6A/6B specific DR probe. NC, negative control. B) U57 copy number normalized by RPP30. The bar plot shows the normalized copy number (CN) for each individual determined by 6A/6B specific U57 probe. NC, negative control. (TIFF) [file pgen.1008915.s006.tiff]
